# Phylogeny of Courtship and Male-Male Combat Behavior in Snakes

**DOI:** 10.1371/journal.pone.0107528

**Published:** 2014-09-24

**Authors:** Phil Senter, Shannon M. Harris, Danielle L. Kent

**Affiliations:** Department of Biological Sciences, Fayetteville State University, Fayetteville, North Carolina, United States of America; Leibniz-Institute of Freshwater Ecology and Inland Fisheries, Germany

## Abstract

**Background:**

Behaviors involved in courtship and male-male combat have been recorded in a taxonomically broad sample (76 species in five families) of snakes in the clade Boidae + Colubroidea, but before now no one has attempted to find phylogenetic patterns in such behaviors. Here, we present a study of phylogenetic patterns in such behaviors in snakes.

**Methodology/Principal Findings:**

From the literature on courtship and male-male combat in snakes we chose 33 behaviors to analyze. We plotted the 33 behaviors onto a phylogenetic tree to determine whether phylogenetic patterns were discernible. We found that phylogenetic patterns are discernible for some behaviors but not for others. For behaviors with discernible phylogenetic patterns, we used the fossil record to determine minimum ages for the addition of each behavior to the courtship and combat behavioral repertoire of each snake clade.

**Conclusions/Significance:**

The phylogenetic patterns of behavior reveal that male-male combat in the Late Cretaceous common ancestors of Boidae and Colubridae involved combatants raising the head and neck and attempting to topple each other. Poking with spurs was added in Boidae. In Lampropeltini the toppling behavior was replaced by coiling without neck-raising, and body-bridging was added. Phylogenetic patterns reveal that courtship ancestrally involved rubbing with spurs in Boidae. In Colubroidea, courtship ancestrally involved chin-rubbing and head- or body-jerking. Various colubroid clades subsequently added other behaviors, e.g. moving undulations in Natricinae and Lampropeltini, coital neck biting in the Eurasian ratsnake clade, and tail quivering in *Pantherophis*. The appearance of each group in the fossil record provides a minimum age of the addition of each behavior to combat and courtship repertoires. Although many gaps in the story of the evolution of courtship and combat in snakes remain, this study is an important first step in the reconstruction of the evolution of these behaviors in snakes.

## Introduction

Many reptilian behaviors are hardwired and stereotyped and therefore heritable [1]. They can therefore be treated as evolutionary characters and mapped onto cladograms to find phylogenetic patterns in behavior, so as to reconstruct the evolution of behavior in a clade. This method has been applied to feeding behavior and defensive displays in snakes [2], but before now it has not been applied to snake courtship or combat behavior.

Courtship and male-male combat in snakes tend to follow ritualistic patterns, and a few reviews of such behavior patterns in snakes have been published [3]–[6]. Until recently, phylogenetic relationships among many snake genera were unknown, so phylogenetic patterns of these behaviors could not be analyzed. Now, however, recent phylogenetic studies of snake genes [7]–[9] have sufficiently clarified relationships to enable such analysis.

Previous authors have recorded data on courtship and male-male combat in 76 species of snakes, all within the clade Boidae + Colubroidea (Table 1). Although the sample includes less than 4% of the species in this very speciose clade, the sample's taxonomic coverage is sufficiently broad to elucidate phylogenetic trends in courtship and combat behaviors. We therefore undertook to identify any such trends.

**Table 1:** Information sources for courtship and combat behavior in snakes.

**Boidae: Boinae**	
	*Candoia bibroni* [37]
	*Corallus caninus* [37]
	*Epicrates striatus* [38]
	*Eunectes murinus* [39]
	*Lichanura trivirgata* [40]
	*Sanzinia madagascariensis* [41]
	*Ungaliophis continentalis* [42]
Boidae: Pythoninae	
	*Morelia boeleni* [37]
	*Morelia spilota* [26], [27]
	*Python curtus* [3]
	*Python molurus* [43]
Colubridae: Colubrinae	
	*Boiga irregularis* [44]
	*Chironius bicavitatus* [45]
	*Chironius flavolineatus* [46]
	*Coluber constrictor* [47]
	*Coronella austriaca* [3]
	*Drymarchon corais* [48]
	*Elaphe quatuorlineata* [3]
	*Lampropeltis alterna* [37]
	*Lampropeltis calligaster* [49]
	*Lampropeltis getula* [50]–[52]
	*Lampropeltis triangulum* [53]
	*Masticophis lateralis* [54]
	*Masticophis taeniatus* [25]
	*Opheodrys aestivus* [55]
	*Pantherophis allegheniensis* [56]
	*Pantherophis guttatus* [56]
	*Pantherophis obsoletus* [56]
	*Pantherophis spiloides* [56], [57]
	*Pantherophis vulpinus* [56], [57], [58]
	*Pituophis catenifer* [4], [59]–[61]
	*Pituophis melanoleucus* [4]
	*Ptyas mucosa* [3]
	*Sonora semiannulata* [62]
	*Spalerosophis diadema* [63]
	*Zamenis longissimus* [3]
Colubridae: Dipsadinae	
	*Farancia abacura* [64]
	*Hydrodynastes gigas* [65]
	*Philodryas baroni* [66]
	*Philodryas olfersii* [67]
Colubridae: Natricinae	
	*Nerodia rhombifera* [3]
	*Nerodia sipedon* [68]
	*Regina septemvittata* [69]
	*Storeria dekayi* [70]
	*Thamnophis butleri* [71]
	*Thamnophis radix* [70]
	*Thamnophis sirtalis* [72]–[75]
Elapidae	
	*Austrelaps superbus* [76]
	*Dendroaspis polylepis* [77], [78]
	*Laticauda colubrina* [79]
	*Pseudechis porphyriacus* [80]
	*Pseudechis porphyriacus* [81]
	*Rhinoplocephalus flagellum* [82]
Lamprophiidae: Lamprophiinae	
	*Lamprophis fuliginosus* [83], [84]
Lamprophiidae: Psammophiinae	
	*Rhamphiophis oxyrhynchus* [85]
Lamprophiidae: Pseudoxyrhophiinae	
	*Leioheterodon madagascariensis* [86]
	*Madagascarophis colubrina* [87]
Viperidae: Crotalinae	
	*Agkistrodon bilineatus* [88]
	*Agkistrodon contortrix* [89]
	*A. piscivorus* [90]–[92]
	*Bothriechis schlegelii* [93]
	*Crotalus atrox* [94], [95]
	*Crotalus durissus* [96]
	*Crotalus horridus* [97], [98]
	*Crotalus ruber* [97]
	*Crotalus viridis* [70]
	*Crotalus oreganus* [97], [99], [100]
	*Lachesis melanocephala* [101]
	*Lachesis stenophrys* [101]
	*Porthidium godmani* [102]
	*Sistrurus miliarius* [103]
Viperidae: Viperinae	
	*Bitis arietans* [5], [104], [105]
	*Bitis caudalis* [106]
	*Bitis gabonica* [107]
	*Vipera berus* [108]
	*Vipera xanthina* [109]

## Methods

From the literature on courtship and male-male combat behavior in snakes (Table 1) we chose 33 behavioral characters (hereafter abbreviated BC) to analyze. These are described in Table 2. For the “courtship” category we included some BCs that occur simultaneously with copulation, but we ignored postcopulatory BCs.

**Table 2:** Behavioral characters considered in this study.

**B: *Bite***	**One snake bites another.**
BB: *Breeding ball*	Several males coil around one female (term from reference [39]).
BM: *Breeding mass*	Several males press onto one female, without coiling.
Bnc: *Bounce*	The male uses vertical neck movements to pat the female's neck (term from reference [69]).
BoB: *Body bridge*	Formation of a high vertical arc with a section of the body (term from reference [107]; “writhe-bump” of reference [56]).
C: *Coil*	One snake forms several coils around another, or both coil around each other, at least anteriorly. As used here, *coil* implies a lack of simultaneous *head raise* or *downward push*.
CG: *Cloacal gaping*	The snake widely opens its cloaca (term from reference [41]).
CR: *Chin-rub*	One snake draws its chin along the skin of another.
CS: *Closed-mouth strike*	One snake strikes at another, with its mouth closed.
DWP: *Downward push*	Both snakes have anterior ends elevated, often coiled around each other, and each attempts to push the other toward the ground.
HB: *Head bob*	Dorsoventral head movement occurs.
HR1: *Head raise (type 1)*	The snake raises its head, and only very little of its neck, off the substrate.
HR2: *Head raise (type 2)*	The snake raises its head and much of its anterior body off the substrate.
HS: *Head shake*	Dorsoventral and mediolateral vibration of the head (term from reference [41]).
J1: *Jerk (type 1)*	The snake gives its head a sudden, staccato jerk. We called the following two behaviors *jerk* also, because they seem to differ mainly in magnitude, with each successive type of *jerk* representing an escalation of magnitude.
J2: *Jerk (type 2)*	The snake gives its head and neck a sudden, staccato jerk.
J3: *Jerk (type 3)*	The snake gives a large part of its body a sudden, staccato jerk (includes “forward jerk” of reference [99], “twitch” of reference [79], and “body jerk” of other authors).
LHR: *Lateral head rub*	One snake rubs the side of its head on the side of another's head.
LP: *Lateral punch*	One snake laterally slams a bend in its body against another's body, to loosen tight coils in the other snake.
MG: *Mouth gape*	The snake holds its mouth open.
MN1: *Mounting (type 1)*	One snake presses down on another with its head. We called the following five behaviors *mounting* also, because each type seemed to be a modification of the previous type, with an increase in magnitude or an elaboration added in each successive type.
MN1b: *Mounting (type 1b)*	One snake presses down on another with its head and neck.
MN2: *Mounting (type 2)*	One snake lies atop another, conforming to the same bodily bends.
MN3: *Mounting (type 3)*	One snake lies atop another, with S-shaped bends draped over the other's dorsum (“dorsal body looping” of reference [6]).
MN4: *Mounting (type 4)*	One snake lies atop another, with lateral undulations that move forward (“caudocephalic waves” of reference [6]) or rearward (“cephalocaudal waves” of reference [6]).
MN5: *Mounting (type 5)*	As with type 4 but the mounting snake is upside-down (his dorsum against hers).
SP: *Spur poke*	One snake pokes another with its spurs.
SR: *Spur rub*	One snake rubs another with its spurs.
Sw: *Sway*	The snake sways its neck back and forth with its anterior portion elevated.
TQ: *Tail quiver*	The snake vibrates its tail.
TR: *Tail raise*	The snake raises its tail. This does not include movements involved in cloacal searching by the male, nor assistance with this by the female.
TV: *Tail wave*	The snake slowly waves its tail back and forth.
TW: *Tail whip*	The snake rapidly whips its tail back and forth.

**Figure 1:**
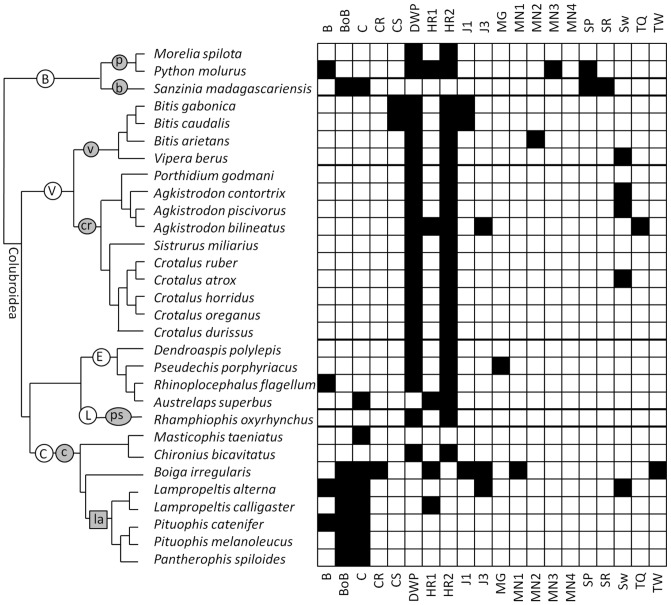
Phylogenetic distributions of behaviors used in male-male combat in snakes, with black boxes representing the recorded presence of a behavior and white boxes representing the absence of a record of the behavior. See Table 1 for references. On the chart on the right, the heavy horizontal lines separate families and subfamilies. On the cladogram, white circles represent families, gray oblongs represent subfamilies, and the gray square is a tribe. Suprageneric taxonomy follows reference [9]. Taxonomic abbreviations: B =  Boidae. b =  Boinae. C =  Colubridae. c =  Colubrinae. cr =  Crotalinae. d =  Dipsadinae. E =  Elapidae. L =  Lamprophiidae. l =  Lamprophiinae. la =  Lampropeltini. n =  Natricinae. P =  Pythoninae. ps =  Psammophiinae. px =  Pseudoxyrhophiinae. V =  Viperidae. v =  Viperinae. For abbreviations of behavioral characters, see Table 2.

**Figure 2:**
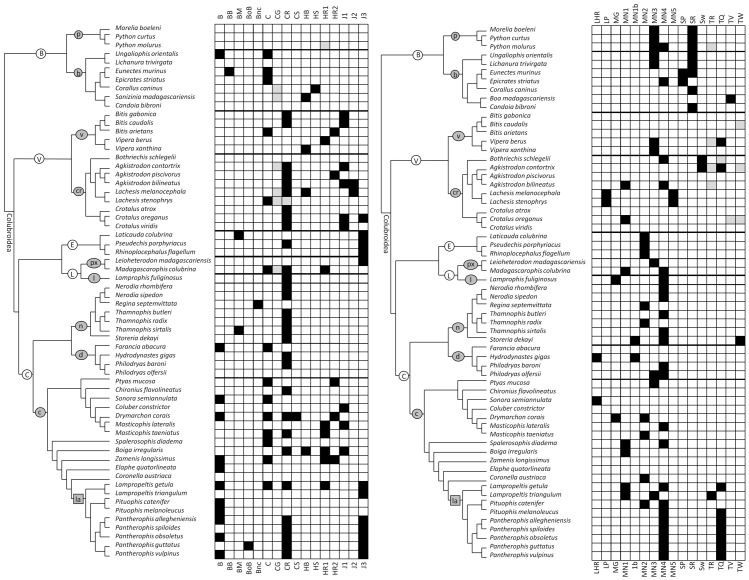
Phylogenetic distributions of behaviors used during courtship and copulation in snakes, with black boxes representing the recorded presence of a behavior in males, gray boxes representing the recorded presence of a behavior only in females, and white boxes representing the absence of a record of the behavior. See Table 1 for references. See the caption to Figure 1 for abbreviations.

Abbreviations on the left are those that are used in Figures 1 and 2.

From recent phylogenetic studies on snakes we created a consensus cladogram of the species in our sample. Onto the consensus cladogram we then mapped each BC (Fig. 1, 2). For Colubroidea we used a recently-published phylogeny [9]. It is missing some of the crotaline species in our sample, so for Crotalinae we used a recently-published phylogeny of Crotalinae [10]. For Boidae we used two recently-published phylogenies [7], [8].

We used outgroup comparison to determine which BCs arose within Serpentes (snakes) and which might have been inherited from a squamate ancestor outside Serpentes and are therefore behavioral symplesiomorphies (ancestral BCs) of Boidae + Colubroidea. Outgroup comparison reveals that many of the behaviors listed above arose within Serpentes, because they are unrecorded in male-male combat and courtship of non-snake squamate (lizard) taxa [1], [11]. BCs that are recorded in lizards include the *bite*, *chin-rub*, *downward push*, *head bob*, *head raise (type 1)*, *jerk (types 1 and 3)*, *mouth gape*, *tail raise*, *tail quiver*, *tail wave*, and *tail whip* [1]. To determine which of these BCs are plausible candidates for behavioral symplesiomorphies of Boidae + Colubroidea, we evaluated the phylogenetic distribution of these behaviors across Squamata, using three previously-published molecular phylogenies of Squamata [12]–[14]. All three phylogenies agree that the successive outgroups to Serpentes are: Iguania + Anguimorpha, Teiidae + (Lacertidae + Amphisbaenia), Scincidae + (Xantusiidae + Cordylidae), and Gekkota.

Of the above BCs, the *bite* and the *mouth gape* and are widespread through all four lizard outgroups [1] and are therefore plausible candidates for behavioral symplesiomorphies of Boidae + Colubroidea. The other BCs are too limited or unclear in phylogenetic distribution to be plausible candidates. The *head bob* is common in Iguania, Lacertidae, and Scincidae [1], [15], [16], but shows no clear phylogenetic pattern across Squamata. The *head raise* is known only in a few iguanians and gekkotans [1]. The *chin-rub* and *downward push* are found mainly in Varanidae, the *jerk (type 1)* in Chamaeleonidae and Varanidae, the *jerk (type 3)* in Chamaeleonidae and Scincidae, the *tail raise* in Iguania, the *tail quiver* in Iguania and Scincidae, the *tail wave* in Iguania and Scincidae, and the *tail whip* in Iguania and Varanidae [1], [17]–[24].

It should be noted that the parameters that define each BC in the eye of an observer may be different from the parameters established by the genes governing each BC. It is therefore possible that BCs that are genetically different in different species may accidentally have been lumped together as one BC in our study. This caveat should therefore be understood to be present within the definition of each BC in Table 2.

## Results for Combat

For male-male combat in snakes, some BCs are recorded only in one species (*chin-rub*, *head-raise type 1*, *mounting [all types]*, *mouth gape*, *spur rub*, *tail quiver*, *tail whip*). Some other BCs show no clear phylogenetic trend, because they are recorded only in two or a few species that are phylogenetically disparate (*bite*, *jerk type 3*, *mount type 2*) (Fig. 1, 2). The *bite* and *mouth gape* are rare and phylogenetically erratic. There is therefore no support for the hypothesis that either is a behavioral symplesiomorphy of male-male combat for Boidae + Colubroidea.

For the other BCs, phylogenetic trends are discernible in at least some snake clades. The results for those BC are as follows.

### Body bridge

This behavior is ubiquitous in Lampropeltini and is therefore ancestral for the clade. It also occurs in *Boiga irregularis*. It was not observed in the other colubrine in our combat sample, *Masticophis taeniatus*, but the *M. taeniatus* combatants fell down a hill during combat [25]; it is possible that a *body bridge* would have occurred if the combat had not thus been disrupted. It is therefore possible that the *body bridge* is ancestral for Colubrinae.

### Coil

The *coil* (without simultaneous *downward push*) is rare outside Colubrinae but present in four of the five sampled colubrine genera. It is more parsimonious to infer that it is ancestral for Colubrinae and has possibly been lost in *Lampropeltis* (a two-step scenario) than to infer that it arose independently in *Masticophis*, *Boiga*, and *Pantherophis* + *Pituophis* (a three-step scenario).

### Closed-mouth strike

This BC is present in the closely-related species *Bitis gabonica* and *B. caudalis*, which therefore may have inherited it from a common ancestor (a one-step scenario) rather than having acquired it independently (a two-step scenario).

### Downward push

This behavior is almost universal outside Colubrinae and is absent within Lampropeltini. It is therefore most likely ancestral for Boidae + Colubroidea and lost in Lampropeltini. Even if it is truly absent in the non-lampropeltine species in our sample for which it has not been observed, it is more parsimonious to infer that it was ancestral for Boidae + Colubroidea and lost multiple times (a four-step scenario) than to infer that the *downward push* was independently acquired in each clade for which all members in our sample exhibit it (a seven-step scenario).

### Head raise (type 2)

The phylogenetic distribution of this BC nearly matches that of the *downward push*, and the two generally occur simultaneously during combat. As with the *downward push*, then, this behavior is most likely ancestral for Boidae + Colubroidea and lost in Lampropeltini. Even if it is truly absent in the non-lampropeltine species in our sample for which it has not been observed, it is more parsimonious to infer that it was ancestral for Boidae + Colubroidea and lost multiple times (a three-step scenario) than to infer that the *downward push* was independently acquired in each clade for which all members in our sample exhibit it (a four-step scenario).

### Jerk (type 1)

This BC is present in the closely-related species *Bitis gabonica* and *B. caudalis*, which therefore may have inherited it from a common ancestor (a one-step scenario) rather than having acquired it independently (a two-step scenario).

### Spur poke

This BC is documented in boas and one python, and may be ancestral for Boidae. It has not been reported in the other python in the sample (*Morelia spilota*), but for one observer the view of the posterior ends of the combatant pythons was obscured by water [26], and for the other observer the view of the posterior ends of the combatant pythons may have been obscured by coils [27]. If the *spur poke* is truly absent in *M. spilota*, it is equally parsimonious to infer that it is ancestral for Boidae and lost in *M. spilota* or that it was independently acquired in the other two boids in the sample. Both scenarios are two-step scenarios.

### Sway

This BC is recorded in three crotalines, a viperine, and one colubrine. No clear phylogenetic pattern is present, but its presence in several members of Viperidae raises the possibility that it is characteristic of Viperidae but has not been explicitly described in other vipers. More research is necessary to determine this. If it is truly absent in the viper species for which it is unrecorded, then it is more parsimonious to infer that it was independently acquired multiple times (a four-step scenario) than to infer that it was independently lost multiple times (an eight-step scenario).

## Results for Courtship

For snake courtship, some BCs are recorded only in one species (*breeding ball*, *body bridge*, *bounce*, *closed-mouth strike*, *head shake*, *tail raise*, *tail wave*, *tail whip*). Some other BCs show no clear phylogenetic trend because they are recorded only in two or a few species that are phylogenetically disparate (*breeding mass*, *head bob*, *lateral head rub*, *mouth gape*, *mounting type 1b*, *sway*) (Fig. 1, 2). The *bite* and *mouth gape* are rare and phylogenetically erratic; there is therefore no support for the hypothesis that either is a behavioral symplesiomorphy for courtship in Boidae + Colubroidea.

For the other BCs, phylogenetic trends are discernible in at least some snake clades. The results for those BC are as follows.

### Bite

Coital neck biting is prevalent in the Eurasian ratsnake clade *Zamenis* + (*Elaphe* + (*Coronella* + Lampropeltini)) and is likely ancestral for the group. It is undocumented in four species in our sample from this clade, but even if it has been lost in those four species, a single-origin scenario is more parsimonious (five evolutionary steps) than a scenario in which coital biting appeared convergently in each taxon within this clade (a seven-step scenario). Outside this clade, documented coital neck biting is rare and has no discernible phylogenetic pattern.

### Chin-rub

This behavior is undocumented in Boidae but is widespread enough in Colubroidea to infer that it is ancestral for Colubroidea. It usually occurs early in courtship, and its lack of documentation in some species may be due to some observers' having arrived after courtship had already begun. Even if the *chin-rub* is truly absent in the species in our sample for which it is undocumented, it is more parsimonious to infer that it is ancestral for Colubroidea and was lost multiple times (a 17-step scenario) than to infer that it was independently gained in each clade in which it is documented in all members (a 21-step scenario).

### Coil

This behavior is documented in every family examined here except Elapidae. It may be ancestral for Boidae + Colubroidea, but its documented phylogenetic distribution is too sparse to be certain. If it is truly absent in the species for which it is undocumented, then it is more parsimonious to infer that it arose independently multiple times (a 13-step scenario) than to infer that it was present in the common ancestor of Boidae + Colubroidea and was lost multiple times (24-step scenario).

### Cloacal gaping

This behavior is present in females of both sampled species of *Lachesis* and may have been inherited from a common ancestor (a one-step scenario) instead of independently acquired (a two-step scenario). Its known phylogenetic distribution outside *Lachesis* is too sparse to draw further phylogenetic inferences.

### Head raise (types 1 and 2)

*Head raising*, of which the two types can be considered to differ mainly in magnitude, is present mainly in Colubrinae. Its recorded distribution is too erratic to determine whether it is ancestral for the subfamily and has been lost in species in which it is unrecorded (a nine-step scenario) or has appeared convergently several times (a six-step scenario). The latter scenario is more parsimonious, but it is also possible that is some species it occurs but has not been observed.

### Jerk (types 1, 2, and 3)

The *jerk*, of which the three types can be considered to differ mainly in magnitude, is unrecorded in Boidae and in the clade Natricinae + Dipsadinae but is prevalent in Viperidae, Elapidae, and Colubrinae and is recorded in one lamprophiid. It is therefore probably ancestral for Colubroidea and has been lost in Natricinae + Dipsadinae. The elevation of its magnitude to *type 3* is universal in the sampled elapids. Because those three species phylogenetically bracket the Australasian elapid clade [9], *type 3* is probably ancestral for that clade but cannot be inferred to be ancestral for Elapidae as a whole. *Type 3* is also present in the one lamprophiid for which the behavior is recorded, and it is possible that a larger lamprophiid sample would reveal that it is ancestral for the clade Elapidae + Lamprophiidae. *Type 3* is ubiquitous in *Pantherophis*, for which it is therefore probably ancestral. If the *jerk* is truly absent in the species for which is it undocumented, then it is more parsimonious to infer that it has been acquired multiple times (a 12-step scenario if changes in the type of *jerk* are not taken into account; 13 steps otherwise) than to infer that it is ancestral for Colubroidea and has been lost multiple times (a 19-step scenario).

### Lateral punch

This BC is recorded in both sampled species of *Lachesis* and is therefore more likely inherited from a common ancestor (a one-step scenario) than independently acquired (a two-step scenario).

### Mounting (types 1–5)

Although unnecessary for intromission in snakes, *mounting* is nearly universal among the sampled species. It is therefore likely ancestral for Boidae + Colubroidea. However, its magnitude differs widely between taxa. The phylogenetic distribution of *type 1* is too erratic and sparse to infer any phylogenetic pattern except in the two sampled species of *Lampropeltis*, which are more likely to have inherited it from a common ancestor (a one-step scenario) than to have acquired it independently (two steps). *Type 2* is present in all three elapid species in the courtship sample and it is therefore more parsimonious to infer that it is ancestral for Australasian Elapidae (one step) than to infer that the three species acquired it independently (three steps). Outside Elapidae it exhibits no discernible phylogenetic pattern. *Type 3* is present in all sampled members of Pythoninae, *Ungaliophis* + *Lichanura*, and *Vipera*. It may therefore be ancestral for each of those three clades. Elsewhere its phylogenetic distribution is too sparse and erratic to infer a pattern. *Type 4* is widespread in Colubridae but is unrecorded in too many sampled colubrids to confidently infer that it is ancestral for the family. However, its phylogenetic distribution indicates that it is likely ancestral for Natricinae, *Philodryas* and Lampropeltini. It is present in both sampled species of *Philodryas*, which therefore more likely inherited it from a common ancestor (one step) than independently acquired it (two steps). It it is truly absent in the species for which it is unrecorded, then within Natricinae it is more parsimonious to infer that it was lost in those species (three steps) than that it was independently acquired in the others (four steps). In Lampropeltini both inferences involve two evolutionary steps and are therefore equally parsimonious. *Type 5* is recorded in both sampled species of *Lachesis*, which therefore more likely inherited it from a common ancestor (one step) than independently acquired it (two steps).

### Spur poke

This BC is recorded only in the sister genera *Eunectes* and *Epicrates*, which therefore more likely inherited it from a common ancestor (one step) than independently acquired it (two steps).

### Spur rub

This BC is widespread enough in Boidae to infer that it is ancestral for the family. If it is truly absent in the species for which it is unrecorded, then it is more parsimonious to infer that it was lost in those species (three steps) than that it was independently acquired in the boid clades that exhibit it (five steps).

### Tail quiver

This BC is ubiquitous in *Pantherophis*, for which it is therefore probably ancestral. Outside *Pantherophis* its phylogenetic distribution is too sparse to infer a pattern.

## Discussion

**Figure 3:**
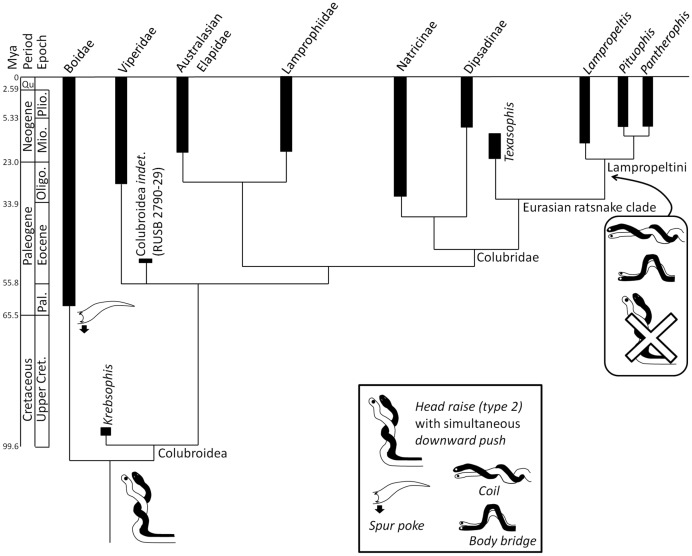
Scenario for the evolution of male-male combat behavior in snakes, based on data presented in Figure 1.

Our results, together with the fossil record, suggest the following scenario for the evolution of male-male combat behavior in snakes (Fig. 3). Its ancestral form for Boidae + Colubroidea included the *head raise (type 2)* with simultaneous *downward push*. This combination of BCs was present by the Cenomanian Age (early Late Cretaceous), the date of the clade's earliest known members [28]. The *spur poke* was added in Boidae by the Paleocene Epoch, the date of the earliest known crown-group boid [29]. The *closed-mouth strike* and *jerk (type 1)* were added in the ancestor of *Bitis gabonica* and *B. caudalis* (date unknown). In Lampropeltini the *downward push* and *head raise (type 2)* were lost and replaced by the *coil* and possibly also the *body bridge* by the mid-Miocene, the date of the earliest known lampropeltine [30].

**Figure 4:**
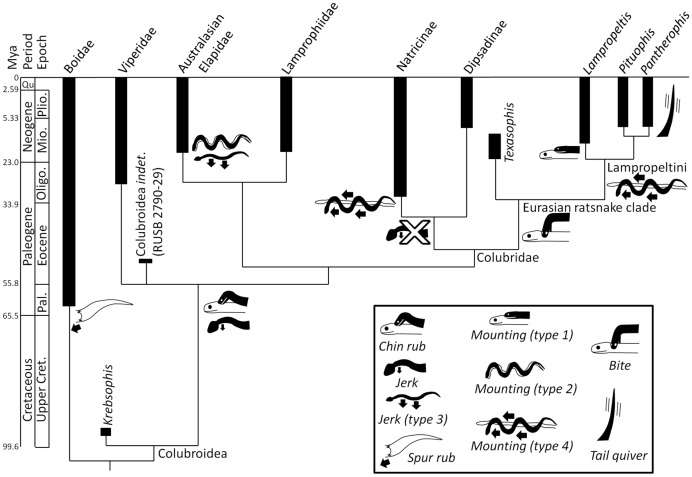
Scenario for the evolution of courtship behavior in snakes, based on data presented in Figure 2.

Our results, together with the fossil record, suggest the following scenario for the evolution of courtship behavior in snakes (Fig. 4). Ancestrally, courtship in Boidae included the *spur rub* by the Paleocene, the date of the earliest known crown-group boid [27]. *Mounting (type 3)* was added in Pythoninae by the early Miocene, the date of the earliest known member of *Python* [31]. It was added in *Ungaliophis* + *Lichanura* by the mid-Eocene, the date of the earliest known member of the latter clade [32]. The *spur poke* was added in *Epicrates* + *Eunectes* by the mid-Miocene, the date of the earliest known member of the clade [33]. Ancestrally, courtship in Colubroidea included the *chin-rub* and *jerk* by the early Eocene, the date of the earliest known crown-group colubroid [34]. *Mounting (type 3)* was added in the ancestor of *Vipera berus* and *V. xanthina* (date unknown); *lateral punch* and female *cloacal gaping* in *Lachesis* (date unknown); *jerk (type 3)* and *mounting (type 2)* in Australasian Elapidae by the early Miocene [35]; loss of *jerk* in Natricinae + Dipsadinae by the early Oligocene [31]; *mounting (type 4)* in Natricinae by the early Oligocene [31], *Philodryas* (date unknown), and Lampropeltini by the mid-Miocene [30]; coital *bite* in the Eurasian ratsnake clade *Zamenis* + (*Elaphe* + (*Coronella* + Lampropeltini)) by the early Miocene [36]; *mounting (type 1)* in *Lampropeltis* by the mid-Miocene [30]; and *tail quiver* in *Pantherophis* by the late Miocene [30].

Often, behaviors that occur in nature are unrecorded. An observer may miss the beginning or end of courtship or combat, a report may omit a behavior that an observer deems too obvious to explicitly state, and captive snake behavior may differ from behavior in the wild. Because of this uncertainty, an inference that a behavior is absent in a clade must be treated with caution. The scenarios presented above are therefore subject to modification pending further data.

Even so, this study shows that available data are sufficient to reveal interesting and important phylogenetic trends in some behavioral characters of combat and courtship in snakes. The results of the study also identify several behavioral characters that need more attention from future research, to help fill current gaps in our ability to reconstruct behavioral evolution in snakes.

This study is an important first step in elucidating the evolutionary history of courtship and combat behavior in snakes. Using available data we have filled in a few important parts of the picture of snake behavioral evolution. To fill in the rest of the evolutionary picture it will be necessary to record observations on combat and courtship behavior in many more snake species. We therefore look forward to further elucidation that future studies will provide.
